# Frontline over ivory tower: key competencies in community-based curricula

**Published:** 2015-04-20

**Authors:** Adam Millar, Janine Malcolm, Alice Cheng, Rebecca Fine, Rene Wong

**Affiliations:** 1 Department of Medicine, University of Toronto, Toronto, Ontario; 2 Deparment of Medicine, University of Ottawa, Ottawa, Ontario

## Abstract

**Background:**

The Royal College of Physicians and Surgeons of Canada mandates that community experiences be incorporated into medicine-based specialties. Presently there is wide variability in community endocrine experiences across Canadian training programs. This is complicated by the paucity of literature providing guidance on what constitutes a ‘community’ rotation.

**Method:**

A modified Delphi technique was used to determine the CanMEDS competencies best taught in a community endocrinology curriculum. The Delphi technique is a qualitative-research method that uses a series of questionnaires sent to a group of experts with controlled feedback provided by the researchers after each survey round. The experts in this study included endocrinology program directors, community endocrinologists, endocrinology residents and recent endocrinology graduates.

**Results:**

Thirty four out of 44 competencies rated by the panel were deemed suitable for a community curriculum. The experts considered the “Manager” role best taught in the community, while they considered the community least suitable to learn the “Medical Expert” competency.

**Conclusions:**

To our knowledge, this is the first time the content of a community-based subspecialty curriculum was determined using the Delphi process in Canada. These findings suggest that community settings have potential to fill in gaps in residency training in regards to the CanMEDS Manager role. The results will aid program directors in designing competency-based community endocrinology rotations and competency-based community rotations in other medical subspecialty programs.

## Introduction

Throughout medicine today there is increased emphasis on the importance of incorporating more of a community focus into postgraduate training programs.^1^, ^2^ However, while felt to be important components of their training, residents have noted inadequacies.^3^ Notably, topics that would be categorized under the CanMEDS Manager role such as office administration and management were the most commonly noted areas of deficiency.^3^ With such gaps in training, and with a large proportion of graduates eventually working outside of academic-based centers, the transition from resident to practicing physician is a challenging period with steep learning curves.^4^ These perceived gaps in training may force new graduates starting their practices outside of academic centers to carry-out “on-the-job” learning concentrated during the initial phases of their careers, creating more anxiety in an already challenging transition period.

The Royal College of Physicians and Surgeons of Canada (RCPSC) has developed national standards for evaluation and accreditation of residency programs across Canada. Its CanMEDS framework ensures that Canadian medical specialist graduates are competent in terms of core knowledge, skills and abilities so that they may meet the needs of patients, communities and society.^5^ The College’s commitment to community-based training is evidenced by Standard 3.1.3 which states: “There should be an integration of teaching resources to include exposure to emergency, ambulatory, and community experiences.”^6^ While the RCPSC defines community learning as taking place outside the conventional teaching service in a teaching hospital,”^7^ community-based rotations in postgraduate training curricula can vary from a rotation in an urban non-academic hospital to a rotation in a rural health care setting. A review by Brown noted that the term “community-based” is often used interchangeably with “decentralized,” “rural” and “distance,”^8^ further adding to this heterogeneity.

The context and environment of the clinical care provided in community hospitals and clinics can be very different to what is provided in academic settings offering additional opportunities for learning. However, the specific competencies that could be developed better in the community (compared to the academic setting) have yet to be reported. In this study, we used a Delphi method to reach consensus among content experts on competencies that could be best learned in a community setting. It is a common and successful method for identifying professional competencies 9 and has been used for planning curricula at the undergraduate and postgraduate levels in several medicine subspecialties.^10^,^11^ Identifying these competencies would enable educators and program directors to better meet the needs of their trainees when designing curricula and community-based experiences.

## Methods

Ethics approval was obtained from the research ethics board of The University of Toronto.

### Participants

Heterogeneous groups with different viewpoints are more likely to produce high quality results in Delphi studies than more homogenous study populations.^12^ Thus a number of key experts across Canada were invited to participate: 1) community-based practicing endocrinologists, 2) endocrinology residency program directors, 3) graduates of endocrinology residency programs within the preceding five years and 4) current endocrinology residents. Endocrinology residency program directors were initially contacted by the research group for consideration of inclusion in the present study. The directors were also asked to suggest present residents, recent graduates or community-based endocrinologists from their region. These individuals in turn were contacted by the research group for consideration of inclusion in the study. One additional advantage of this survey method is that it allowed for consensus opinion to be reached effectively despite wide geographic separation between panel members.

### Design

**Figure 1:**
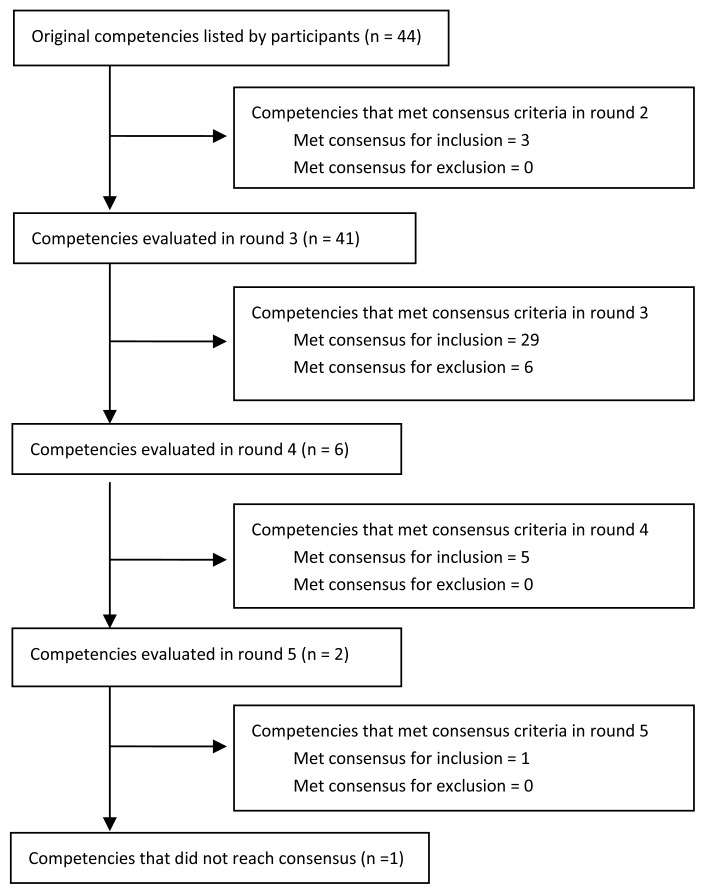
Competencies evaluated in each survey round

The specific steps involved in a Delphi method are outlined in Figure 1. In the first round of the Delphi process, participants were asked to: “Please list the competencies/skills for which you think community endocrine rotations would provide the best learning opportunities over that of an academic centre.”

Responses were collected and stored on SurveyMonkey.^®^ Two (2) of the authors (A.M., R.W.) coded the responses, organized and classified them under the most appropriate CanMEDS roles.

The list was then used to form the basis of a quantitative questionnaire for subsequent rounds. For each objective listed, participants were asked: “Rate the extent to which a community endocrine rotation would provide better learning opportunities over that of an academic centre” on a five-point Likert Scale. The five (5) options were as follows:

1. Not learned in a community rotation (best learned in an academic centre) (score = 1/5)
2. Seldom learned in a community rotation (score = 2/5)
3. Neutral (community rotation equal to an academic centre) (score = 3/5)
4. Better learned in a community rotation (score = 4/5)
5. Best learned in a community rotation (score = 5/5)

Participants were also invited to add suggestions for topics not listed in the questionnaire.

### Data Analysis

Medians, modes and difference between the 25^th^ and 75^th^ percentiles (25^th^–75^th^ percentile) were calculated for each topic. Criteria for consensus were derived from methods reported in prior Delphi studies.^13^–^15^ Consensus was defined if the 25^th^–75^th^ percentile values of the panel’s ratings were equal to or less than one (1). For this round, topics for which consensus was reached, with a median score of five (5), were included as a “Priority 1” topic (“Must be able to”). Conversely, items meeting consensus with medians of one (1)–two (2) were excluded from the final list.

Items that did not meet criteria for inclusion or exclusion, and new items suggested by the panelists were included in the Round 3 questionnaire. Panelists were provided mean scores from the prior round. In this and subsequent rounds, the panel was asked to rate each topic using a four (4)-point Likert scale to avoid non-committal responses. Inclusion of topics was based on the following priority classification criteria (which were adapted from published reports^13^–^15^):

- Topics for which consensus was reached, with a median score of four (4) and a mode of four (4) rated by over 75% of respondents: “priority one consensus.”
- Topics for which consensus was reached, with a median score of 3, with more than 75% of respondents rating it three (3) or four (4): “priority two.”
- Topics for which consensus was reached, with a median score of three (3), with 50–75% of respondents rating it three (3) or four (4): “priority three.”

Items with medians of one (1)–two (2) were excluded from the final list of essential competencies. All other items, including those that did not reach consensus were resubmitted for the next round to be rerated. The process was planned to continue until consensus was reached for all items.

## Results

The community experts in the study included individuals from four (4) distinct groups: six (6) endocrinology program directors, three (3) community endocrinologists, six (6) endocrinology residents and three (3) recent endocrinology graduates (see Table 1). In Delphi Studies it has been noted that larger sample sizes can lead to a greater generation of data. Adequate results, however, can be achieved with sample sizes as low as 15.^16^ Among the original 24 experts that were invited to participate, 15 completed at least four (4) of the five (5) survey rounds. After the first qualitative round, 44 topics were identified by the panel. An additional four survey rounds were required to reach consensus on 43 of the originally listed 44 competencies (see Figure 1). Despite a goal to continue the process until consensus was reached for every item, by the end of the fifth round one topic remained for which consensus had not been reached: “Discuss opportunities to engage in teaching in the community at the undergraduate, postgraduate and continuing health education levels” (Scholar). As participants were openly citing survey fatigue, and the continuation rate of participants was dropping, no further rounds were conducted and ultimately this topic was not included.

Table 2 shows the list of topics identified by the panel for which community settings provide better learning experiences over that of an academic center. Of the original 44 competencies, 34 were deemed essential by the panel (see Table 2). Competencies that reached consensus earliest and were most likely to be considered essential included items from the “Manager” CanMEDS role. The competencies that were rated highest overall included:

Describe what to look for in a potential office space for an outpatient endocrinology practice (Manager
Discuss principles of how to negotiate a lease for office space (Manager)
Describe strategies to advertise your practice to referring physicians when starting a practice (Manager)

Of note, describing what to look for in a potential office space was the only competency rated by the panel that achieved consensus priority one. Furthermore, the majority of the items deemed to be better learned in community fell into the non-Medical Expert roles, with all 16 “Manager,” four (4) “Communicator” and three (3) “Collaborator” competencies deemed to be essential to include in community rotations.

Conversely, “Medical Expert” competencies were least likely to be taught better in community settings, with seven (7) of the nine (9) listed competencies receiving low scores on the 5 point Likert Scale.

## Discussion

This paper reports the consensus opinion of key experts in residency education: program directors, community specialists, recent graduates and current residents, regarding what topics could best be learned in a community-based endocrinology setting. By defining these competencies, it helps define the role of community experiences within broader residency training program. Ultimately this helps ensure that residents meet societal and professional needs that are in line with the mandates of RCPSC in the most effective setting. Classifying these competencies under the CanMEDS roles allows program directors to assist in planning or modification of current community-based curricula and rotations.

To our knowledge this is the first time a Delphi process has been used to define a set of CanMEDS competencies for which community-based learning experiences provide advantages over academic experiences. This study was conducted in the setting of one medical subspecialty, endocrinology; however given that the majority of the identified topics were not specific to endocrinology and focused on non-clinical aspects of specialists’ roles they may be applicable to other fields of medicine. The successful use of the Delphi process in the development of a community-based curriculum in a family medicine training program was demonstrated by Wolff *et al.*^2^ Using this technique, the authors identified key elements to incorporate into a community health curriculum for family medicine residents. The paper by Wolff *et al.* differed from the present study as it focused on a dedicated community health curriculum for Family Medicine residents, while the present study focuses on community-based rotations embedded in pre-existing endocrinology curricula across Canada.

In a study by Garfunkel *et al.*, a panel identified eight key competencies for a community-based pediatrics curriculum 17 Most of these competencies would be considered “advocacy” or “communicator” roles if they were classified using the CanMEDS framework. Similar to Garfunkel *et al.*’s paper, the present study identified four (4) key competencies in both the advocacy and communicator roles that could be better learned in community settings. Our study differed, in that it identified 34 key competencies, including 16 from the “manager” role alone. It also added several “manager” competencies not mentioned in Garfunkel’s study, such as knowing what to look for in a potential office space, the principles of hiring support staff, demonstration of effective time management and appropriate billing practices.

The present study contributes to the literature on competency-based education, specifically in regards to the CanMEDS “Manager” role, for which the most comparable ACGME competency is “Systems-based learning.” The teaching of these competencies has been widely noted to be deficient across several Canadian training programs,^3^, ^18^ and American training programs.^19^,^20^ The results from the present study further contribute to the existing literature on the “Manager” role by showing that many believe community experiences could have a role in exposing residents to opportunities to develop expertise in this role, above and beyond what they could get in more common academic-based settings. The final list of topics derived from this study also provides specific examples of competencies under this role such as seeking out office space, negotiating leases for offices and advertising of medical practices to the surrounding community.

It was surprising that five (5) survey rounds were required to reach consensus on most of the competencies listed in this paper. Furthermore, it was unexpected that after five (5) rounds, one competency would still not reach consensus among the participants. Delphi studies usually only require a few rounds to reach consensus, and prior studies have suggested that little change occurs after the second or third rounds.^21^ The fact that our study did not reach consensus as quickly may be a function of the diverse expert groups involved in the study. Having several different expert groups likely strengthened our results as it led to opinions that were more in line with the opinions of the discipline as a whole.

Now that the most essential CanMEDS competencies have been defined, the next step is to use the information from the present study towards faculty development, specifically on how to most effectively teach these competencies in community rotations. Additional work will also be required on how to best evaluate these competencies in a community setting, given that the CanMEDS “Manager” role has been viewed as a difficult skill to adequately evaluate.^22^ Review of the final list of competencies shows that the conclusions from this study would likely be generalizable to other medical subspecialties that have significant ambulatory patient exposure. Future studies may look at whether patients and family physicians would agree with the list of competencies listed in the present study, thereby providing a more comprehensive view on what competencies are viewed as best taught in the community.

As this was a cross sectional study, the opinions offered were valid only for a defined moment in time. Over time, opinion may change as to what competencies are most important to include in community-based endocrinology rotations. Additionally, the Delphi Process is designed to favor consensus opinions, thus it tends to minimize the impact of opinions that may be held by a minority. Perhaps the largest limitation to the Delphi technique is that there are no universal guidelines to assist in its use as a research tool.^23^ As the Delphi requires commitment to several rounds of interviewing, there are risks of participant dropout at various stages of information gathering. We were fortunate that even despite participant dropout, we were able to still achieve a final sample size of 15 participants, which has been shown to be an effective number in Delphi studies.^16^ In fact, it has been suggested that sample sizes greater than 30 typically do not result in improved validity,^24^ and may only serve to complicate the process. Another potential limitation of the present study is the exclusive use of endocrinologists or endocrine trainees in the survey. Use of other community-based physicians that treat endocrine conditions (such as internal or family medicine) may have added a different viewpoint to the findings presented in this paper. Finally, the present study was limited by the fact that consensus was not achieved on one of the selected competencies, despite four rounds of quantitative surveys.

## Key Learning Points

- In Canada, community experiences are mandated by The Royal College of Physicians and Surgeons, but their role in residency training is not well defined.
- What constitutes a “community experience” varies greatly among training centres
- Community settings can provide learning experiences above that provided in traditional academic settings.
- Community settings have potential to fill in gaps in residency training in regards to the CanMEDS Manager role
- Further studies are needed to determine how best to teach and assess specific defined competencies under the Manager Roles in community rotations

## Figures and Tables

**Table 1:** Characteristics of initial participants in study

Characteristic	Description and number of representatives
Job description	Endocrinology Program Director: 6 Community-based endocrinologist: 3 Recent endocrinology graduate: 3 Endocrinology resident: 6
Geographic distribution	Western Canada: 4 Ontario: 11 Quebec: 3 Eastern Canada: 0

**Table 2:** Final list of 34 competencies created by key experts (with consensus levels in parentheses). Consensus levels are as follows: Priority 1: Median of 5, with a mode of 5 rated by over 75% of respondents. Priority 2: Median of 4, with > 75% of respondents rating it 4 or 5. Priority 3: Median of 4, with 50–75% of respondents rating it 4 or 5

**MANAGER** Discuss principles of recruiting, hiring and managing support staff personnel (e.g. nurses, assistants, secretaries, etc.) (2) Demonstrate appropriate billing practices, including criteria for commonly-used billing codes (2) Design an efficient office schedule (2) Describe strategies to balance time between professional activities, including inpatient and outpatient responsibilities (2) Describe strategies to effectively balance time between professional and personal/home life (3) Compare the benefits and drawbacks of different patient charting options (such as electronic vs. paper based systems) (2) Determine appropriate time period(s) to arrange follow-up appointments (2) Demonstrate how to effectively follow up lab and test results in a time-appropriate manner, based on urgency (3) Demonstrate how to respond appropriately to lab and test results of differing urgencies i.e. do you call them with results? Do you rebook them within a week? How are abnormal labs flagged and dealt with? (3) Describe strategies to locate and utilize community resources to help optimize patient care (2) Discuss principles of how to negotiate a lease for office space (2) Describe what to look for in a potential office space for an outpatient endocrinology practice (1) Describe strategies to advertise your practice to referring physicians when starting a practice (2) Discuss principles of dealing with patients who do not show up for scheduled appointments (in terms of documentation and charging patients) (2) Recognize and evaluate administrative opportunities within community hospital or government settings (3)? Demonstrate how to appropriately bill for uninsured services (2)?
**HEALTH ADVOCATE** Discuss strategies to create and tailor programs to meet the needs of the surrounding community (e.g. language or cultural needs) (2) Describe how to engage in local advocacy work for the surrounding community (2) Discuss different strategies for advocating for your patients (for example, those in financial need or with special needs) (3) Complete the steps required to request coverage for specific drugs not routinely covered by provincial health care plans (3)
**COMMUNICATOR:** Create an effective consultation letter to the referring physician in an efficient manner (3) Provide advice to another health care provider (e.g. primary care physician) via telephone consultation (3) Effectively discuss coordination of care or shared care of a patient with his/her other care provider (e.g. primary care physician) (3) Describe situations for which urgent communication with a patient’s primary care physician is appropriate (3)
**COLLABORATOR:** Effectively collaborate with allied health professionals outside of the office setting (2) Describe how to locate allied health professionals in the community with whom to collaborate in patient management (2) Describe how to set up and/or engage in a network of endocrinologists and health care professionals in the community to support lifelong learning and continuing professional development (2)
**MEDICAL EXPERT:** Demonstrate medical expertise in the recognition and management of common and uncommon presentations of common endocrine problems (3) Describe how to deal with urgent outpatient issues (3)
**SCHOLAR** Describe strategies, opportunities and methods to promote lifelong learning in a community setting (2) Describe how to accommodate medical students and residents in a community practice (2) Describe how to locate mentorship opportunities in the community setting (2)
**PROFESSIONAL** Discuss strategies to promote ethical practice when interacting with representatives from the pharmaceutical industry (3) Describe how to appropriately end a physician-patient relationship (3)

## References

[1] Morrison J (2006). Learning in teaching hospitals and the community: time to get the balance right. Med Educ.

[2] Wolff M, Hamberger LK, Ambuel B (2007). The development and evaluation of community health competencies for family medicine. WMJ.

[3] Card SE, Snell L, O’Brien B (2006). Are Canadian general internal medicine training program graduates well prepared for their future careers?. BMC Med Educ.

[4] Smith LG (1996). First year of practice: a year of rapid learning. Acad Med.

[5] The Royal College of Physiciansand Surgeons of Canada http://www.royalcollege.ca/public/resources/aboutcanmeds.

[6] Bandiera G, Sherbino J, Frank JR (2006). The CanMEDS Assessment Tools Handbook an Introductory Guide to Assessment Methods for the CanMEDS Competencies.

[7] Canada TRCoPaSo http://rcpsc.medical.org/residency/accreditation/positionpapers/Community_Learning_Experiences_e.pdf.

[8] Brown A (2008). Community-based education: brief review. Future of Medical Education in Canada Project - Undergraduate Medical Education Environmental Scan Project.

[9] Dunn WR, Hamilton DD, Harden RM (1985). Techniques of identifying competencies needed of doctors. Med Teach.

[10] Lazarou J, Hopyan J, Panisko D, Tai P (2011). Neurology for internal medicine residents: working towards a national Canadian curriculum consensus. Med Teach.

[11] Penciner R, Langhan T, Lee R, McEwen J, Woods RA, Bandiera G (2011). Using a Delphi process to establish consensus on emergency medicine clerkship competencies. Med Teach.

[12] Murphy MK, Black NA, Lamping DL (1998). Consensus development methods, and their use in clinical guideline development. Health Technol Assess.

[13] McKenna HP (1994). The Delphi technique: a worthwhile research approach for nursing?. J Adv Nurs.

[14] de Oliveira Filho GR, Schonhorst L (2004). The development and application of an instrument for assessing resident competence during preanesthesia consultation. Anesth Analg.

[15] Paterson Davenport LA, Hesketh EA, Macpherson SG, Harden RM (2004). Exit learning outcomes for the PRHO year: an evidence base for informed decisions. Med Educ.

[16] Fiander M, Burns T (1998). Essential components of schizophrenia care: a Delphi approach. Acta Psychiatr Scand.

[17] Garfunkel LC, Sidelinger DE, Rezet B, Blaschke GS, Risko W (2005). Achieving consensus on competency in community pediatrics. Pediatrics.

[18] Lieberman L, Hilliard RI (2006). How well do paediatric residency programmes prepare residents for clinical practice and their future careers?. Med Educ.

[19] Martin GJ, Curry RH, Yarnold PR (1989). The content of internal medicine residency training and its relevance to the practice of medicine: implications for primary care curricula. J Gen Intern Med.

[20] Cantor JC, Baker LC, Hughes RG (1993). Preparedness for practice: young physicians’ views of their professional education. JAMA.

[21] Crisp J, Pelletier D, Duffield C, Nagy S, Adams A (1999). It’s all in a name. When is a ‘Delphi study’ not a Delphi study?. Aust J Adv Nurs.

[22] Chou S, Cole G, McLaughlin K, Lockyer J (2008). CanMEDS evaluation in Canadian postgraduate training programmes: tools used and programme director satisfaction. Med Educ.

[23] Hasson F, Keeney S, McKenna H (2000). Research guidelines for the Delphi survey technique. J Adv Nurs.

[24] de Villiers MR, de Villiers PJ, Kent AP (2005). The Delphi technique in health sciences education research. Med Teach.

